# Does the terrain influence running critical power and biomechanics? An in-field study with highly trained trail runners

**DOI:** 10.1007/s00421-025-05840-z

**Published:** 2025-07-13

**Authors:** Diego Jaén-Carrillo, Santiago A. Ruiz-Alias, Felipe García-Pinillos

**Affiliations:** 1 Department of Sport Science, University Innsbruck, Innsbruck, Austria; 2 Department of Physical Education and Sports, Faculty of Sport Sciences, Sport and Health University Research Institute (iMUDS), University of Granada, Cam. de Alfacar, 21, Norte, 18071 Granada, Spain; 3 Department of Physical Education, Sport and Recreation, Universidad de La Frontera, Temuco, Chile

**Keywords:** Biomechanics, Modelling, Off road, Performance, Running

## Abstract

This study aimed to assess the effects of three flat running surfaces (i.e. athletic track, road, and gravel) on the critical power (CP) parameters and running patterns of highly trained trail runners. Within a two-week timeframe, thirteen male and seven female trail runners underwent three testing sessions to evaluate CP and the work over CP (*W*’). Each session comprised two time trials of 9 and 3 minutes, separated by a 30-min rest, in which a Stryd running power meter was used to collect the data. The CP and *W*’ were subsequently determined using the inverse of time linear CP model. There were no significant differences in CP across the different surfaces (*F*_(2,38)_= 1.4; *p* = 0.253). However, significant differences were found in *W*’ (*F*_(2,38)_= 3.8; *p* = 0.032). Specifically, athletes displayed a higher *W*’ on the track compared to gravel (1.8 [0.2 to 3.4] kJ, *p =* 0.026), and higher, though non-significant, *W*’ on the road compared to gravel (0.9 [−0.7 to 2.5] kJ, *p* = 0.478). Regarding the running patterns, the athletes displayed lower duty factor on the track compared to gravel (−1.1 [−2.2 to −0.1] %; *p* = 0.030) as well as on the road compared to gravel (−1.1 [−2.0 to −0.1] %; *p =* 0.019). In conclusion, the CP remained stable across surfaces, whereas W’ was reduced on gravel compared to track and road. The differences in W’ were accompanied by significant changes in the athletes’ running patterns. Specifically, athletes exhibited a lower duty factor on the track and road compared to gravel, resulting in a more aerial running form.

## Introduction

Trail running is commonly described as a running event held in natural settings, with minimal inclusion of paved or asphalt surfaces, which do not make up more than 20% of the entire course (Discover Trail Running 2024). This form of endurance race unfolds across a diverse range of natural landscapes, including mountains, forests, rural areas, and deserts, featuring courses that vary significantly in terrain, occurring on terrains that have uphill, downhill, and flat segments. Distances can span from short runs of just a few kilometres to ultra-trail events exceeding 80 kilometres, without restrictions on the amount of elevation gained or lost (Discover Trail Running 2024). For instance, one event of the Trail World Championship in 2023 covered a distance of 45.2 km and included an elevation gain of 3250 metres (World Mountain and Trail Running Championships Innsbruck-Stubai 2023). Over the last two decades, trail running has witnessed an exponential growth of 2394% in participants, with a 231% increase observed in the last decade alone, based on data from 2022 (Andersen 2022). The recognition of trail running by World Athletics in 2015 as an official discipline of athletics (Running 2024) has significantly contributed to its rapid development and professionalization.

To enhance performance, coaches and athletes regularly engage in testing and monitoring as integral components of their daily routines, aiming to gain insights into the adaptations arising from training. Within the array of metrics employed, the concept of critical power (CP) is particularly noteworthy for its pragmatic application. This is because it simplifies the complex task of identifying specific physiological capacities, such as the highest intensity at which metabolic equilibrium is maintained (i.e. CP) and the work capacity beyond this threshold (i.e. W'), through straightforward, non-invasive methods (Jones and Vanhatalo 2017). In trail running, CP is monitored via running power, which shows strong consistency and alignment with external work and oxygen consumption across diverse settings (e.g. indoor vs outdoor) and varying conditions (e.g. speed, body weight, and incline) (García-Pinillos et al. 2019; Imbach et al. 2020; Cerezuela-Espejo et al. 2021; Taboga et al. 2021; Ruiz-Alias et al. 2022). However, to the authors' knowledge, CP and W' in trail running have received very little attention.

Prior to determining and setting training intensities through running CP, athletes must undergo a CP test (Ruiz-Alias et al. 2022). This evaluation can be conducted both on a treadmill and in outdoor settings, such as on an athletic track or a paved road among other running surfaces. Previous research comparing CP values obtained from treadmill versus athletic track assessments suggested that the CP values from these two environments might not be directly comparable (^11^). This discrepancy suggests a limitation in directly transferring CP values from treadmill assessments to track environments, or vice versa, with findings indicating notably higher CP values on the track than those recorded on a treadmill (Ruiz-Alias et al. 2023a). Common non-technical surfaces, such as park trails, multi-use single tracks, or forest paths, are frequently utilized by road and trail runners for their daily training activities (Hamill et al. 2022). While road runners often incorporate athletic tracks and paved roads into their weekly training schedules, trail runners also resort to these surfaces when mountain paths are inaccessible, during the off-season, or when seeking high-intensity training. However, the effect of different terrains on CP values, particularly on surfaces like tartan, road, and groomed trails (gravel), remains unclear. Although power calculations of current commercial power meters remain undisclosed, it is expected that running spatiotemporal variables may play a role. Previous studies suggest that variables such as ground contact time (GCT), step frequency, and step length remain largely unchanged across different running surfaces under submaximal steady-state conditions, whereas leg stiffness adapts (Ferris et al. 1999, 1998; Hollis et al. 2019). However, when increasing the running intensity, some significant interactions in GCT and step time have been reported between track and grass surfaces (Hollis et al. 2019).

Given the widespread adoption of the CP variables within the running community for guiding training and racing, a crucial question is whether CP and W’ remain consistent across different common running surfaces (i.e. athletic track, asphalt road, and well-groomed fine gravel) among highly trained trail runners. A secondary objective is to examine how these surfaces influence running patterns. Based on previous research in the field (Ruiz-Alias et al. 2023a; Hollis et al. 2019), it is hypothesized that surface type affects athletes’ running patterns and, consequently, CP-related variables. Additionally, given the potential influence of running surface on leg stiffness (Ferris et al. 1999, 1998), its variation across different surfaces will be analysed. It is hypothesized that leg stiffness will adapt to each surface, exhibiting significantly different values.

## Methods

### Participants

Thirteen male and seven female trail runners (*n* = 20), all competing internationally, volunteered for this study (Table 1). All participants were free from musculoskeletal injuries and cardiopulmonary and metabolic conditions. Written informed consent was obtained after providing both verbal and written explanations of the experimental protocol, ensuring participants were fully aware of the potential risks. An a priori power analysis using G*Power (Faul et al. 2007) was conducted to determine the necessary sample size, assuming a large effect size (*d* = 0.8), *α* = 0.05, and a desired power of 0.80. It was found that 12 participants would be sufficient to detect terrain-related differences, ensuring result validity and robustness. This study included 20 trail runners to account for dropouts (injuries and/or timing with competitions). The ethics committee at the University of Innsbruck approved the study protocol (no. 36/2023), which adhered to the principles outlined in the Declaration of Helsinki. Participants were recruited using convenience and snowball non-probability sampling.

**Table 1.** Demographic description of the participants

	Male	Female
*n*	13	7
Age (years)	29.9 ± 4.9	27.9 ± 1.9
Height (cm)	180.3 ± 6	170.1 ± 5.7
Weight (kg)	71.5 ± 4.3	61.1 ± 7.8
BMI (kg·m^−2^)	22 ± 1.3	21 ± 1.8
TR Experience (years)	5.8 ± 2.9	3.6 ± 1.7
Weekly volume (km)	83.5 ± 31	72.1 ± 36.4
ITRA P.I.^	723 (547–910)	621 (519–700)

*BMI* Body Mass Index, *TR* Trail Running, *ITRA P.I.* The Performance Index offers a means of assessing and contrasting the proficiency of trail runners globally, structured on a scale with a maximum of 1000 points. The upper limit of the scale represents the theoretical best performance (https://itra.run/FAQ/PerformanceIndex)

^Values are shown as mean (min value–max value)

### Experimental design

**Fig. 1:**
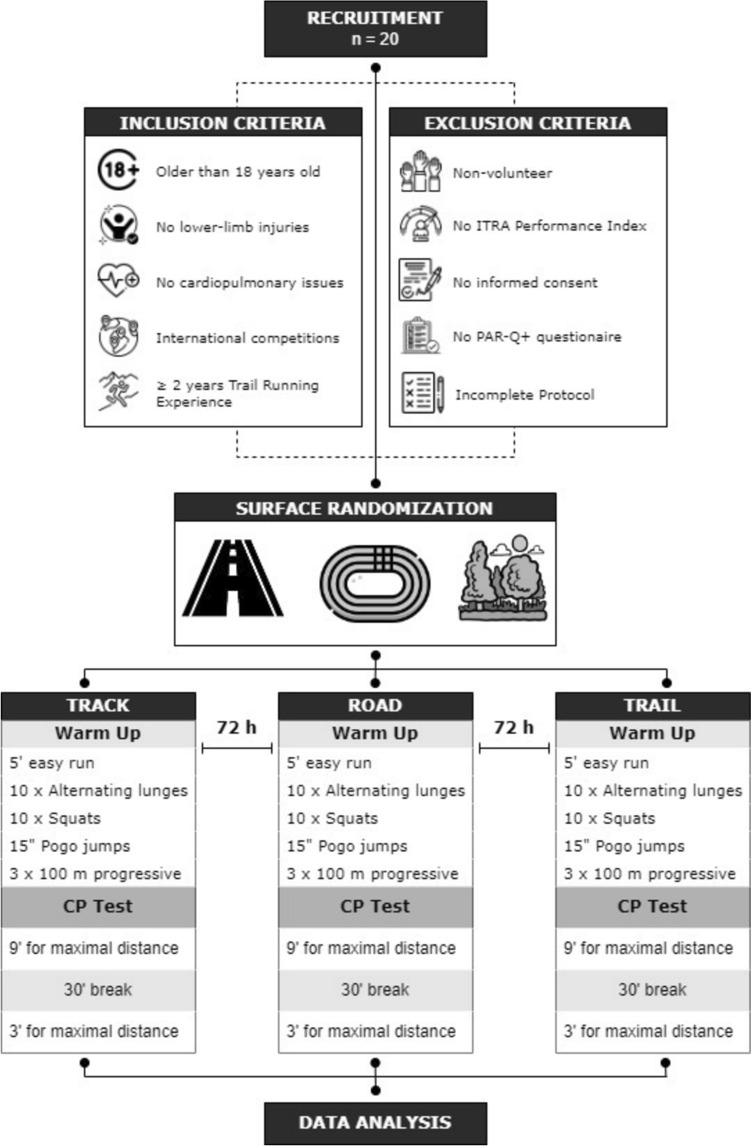
Participants’ recruitment procedure and experimental design

A repeated measures design was utilized to examine the influence of different running terrains. Within a 2-week timeframe, participants underwent three testing sessions to evaluate CP and W' on distinct flat surfaces, including an athletic track, road, and a gravel path made of fine crushed gravel and sand (Figure 1). The assignment of the testing surface was randomized. Each testing session comprised a 9-min maximal effort run followed by a 3-min maximal effort run, separated by a 30-min rest interval (Ruiz-Alias et al. 2022, 2023b). These assessments were conducted on the three different flat surfaces, with each test separated by a 72-hour period. The CP and W' were subsequently determined using the inverse of time linear CP model (Whipp et al. 1982). Participants were instructed to refrain from engaging in vigorous activities (such as running interval sessions) within the 24 hours preceding each test. All testing sessions were conducted under consistent environmental conditions (± 2 °C ambient temperature, ± 1% humidity and ± 1 m/s air velocity), at the same time of the day (± 1 h) and utilizing the participants the same own racing footwear for all the terrains. Although it was not an exclusion criterion, none of the participants used advanced footwear technology.

### CP and running performance variables

Participants initiated the testing sessions with a standardized warm-up, comprising 10-min of running at a low-to-moderate intensity, equivalent to the intensity associated with easy long-running sessions. Following a series of dynamic mobility exercises, the warm-up was concluded with three progressive runs of approximately 100 metres. Athletes were encouraged to cover the maximum distance possible. The configuration of 9- and 3-min time trials was chosen based on the established validity of CP (Ruiz-Alias et al. 2022), the alignment of CP and W' with those determined in a 5-time trial configuration through the same procedure (Ruiz-Alias et al. 2023b), and the reliability of these parameters when considering different protocol configurations (i.e. trials order, day, or within session recovery) (Ruiz-Alias et al. 2023a). The perceived effort following each time trial was evaluated using the Borg CR-10 scale (Borg 1998).

The Stryd power meter (Stryd Next Gen, Boulder, CO, USA) was utilized to calculate the mean absolute power output (W) during each time trial. Additionally, speed and distance covered were also collected using the aforementioned running power meter. Body mass and height were measured using a weight and a height scale, respectively (Seca 813 and Seca 213; Seca Ltd, Hamburg, Germany), during the initial testing session and maintained for subsequent sessions within the power meter. For each testing session, the power meter was clipped to the laces of the right shoe. The Stryd was paired to a Garmin Forerunner 735XT sport watch (Garmin Ltd., Olathe, Kansas, USA). The recorded power outputs (PO) from both the 9- and 3-min trials were graphed within the CP_1/time_ model to determine CP and *W*’ as the point of interception and the slope of the regression line, respectively, following the next equation (Eq. 1) (Whipp et al. 1982):

1 $$PO = W^{\prime } *\left(1/t \right) + {\mathrm{CP}}).$$

### Running pattern and performance variables

During the predicting trials (i.e. 9- and 3-min), the spatiotemporal parameters of flight time (FT), GCT, and cadence, as well as power output, speed, and distance were collected using the Stryd power meter, while duty factor (DF) was calculated by Eq. (2):

2 $$\mathrm{DF} = \left( \left(\frac{\mathrm{GCT}}{\mathrm{GCT} + {\mathrm{FT}}} \right)*100 \right).$$

The validity and reliability of these parameters reported by the Stryd power meter have been reported in previous studies (Imbach et al. 2020; Garcia-Pinillos et al. 2021). Furthermore, leg stiffness was also collected using the Stryd power meter (Imbach et al. 2020).

### Statistical analyses

All values are expressed as mean ± standard deviation (95% confidence intervals). Normal distribution and homogeneity of variances of the data were confirmed by the Shapiro–Wilk test and Levene’s test, and sphericity was examined using Mauchly’s test for all variables. A repeated measures ANOVA was utilized to compare the CP parameters (i.e. CP and *W*’) reported from the different flat terrains (track vs gravel; road vs gravel; track vs road). A two-way repeated measures ANOVA (surfaces × predicting trials [9- and 3-min]) was performed to explore the effects of surface and their interaction on the spatiotemporal parameters. To better interpret the practical significance of the observed differences across surfaces, the Smallest Worthwhile Change (SWC) was calculated for each performance-related variable as 0.2 times the between-subject standard deviation. The level of significance used was *p* < 0.05. Data analysis was carried out using Jamovi software package (version 2.3.26, The Jamovi Project), and statistical significance was set at *p* < 0.05.

## Results

### CP and running performance variables

**Fig. 2:**
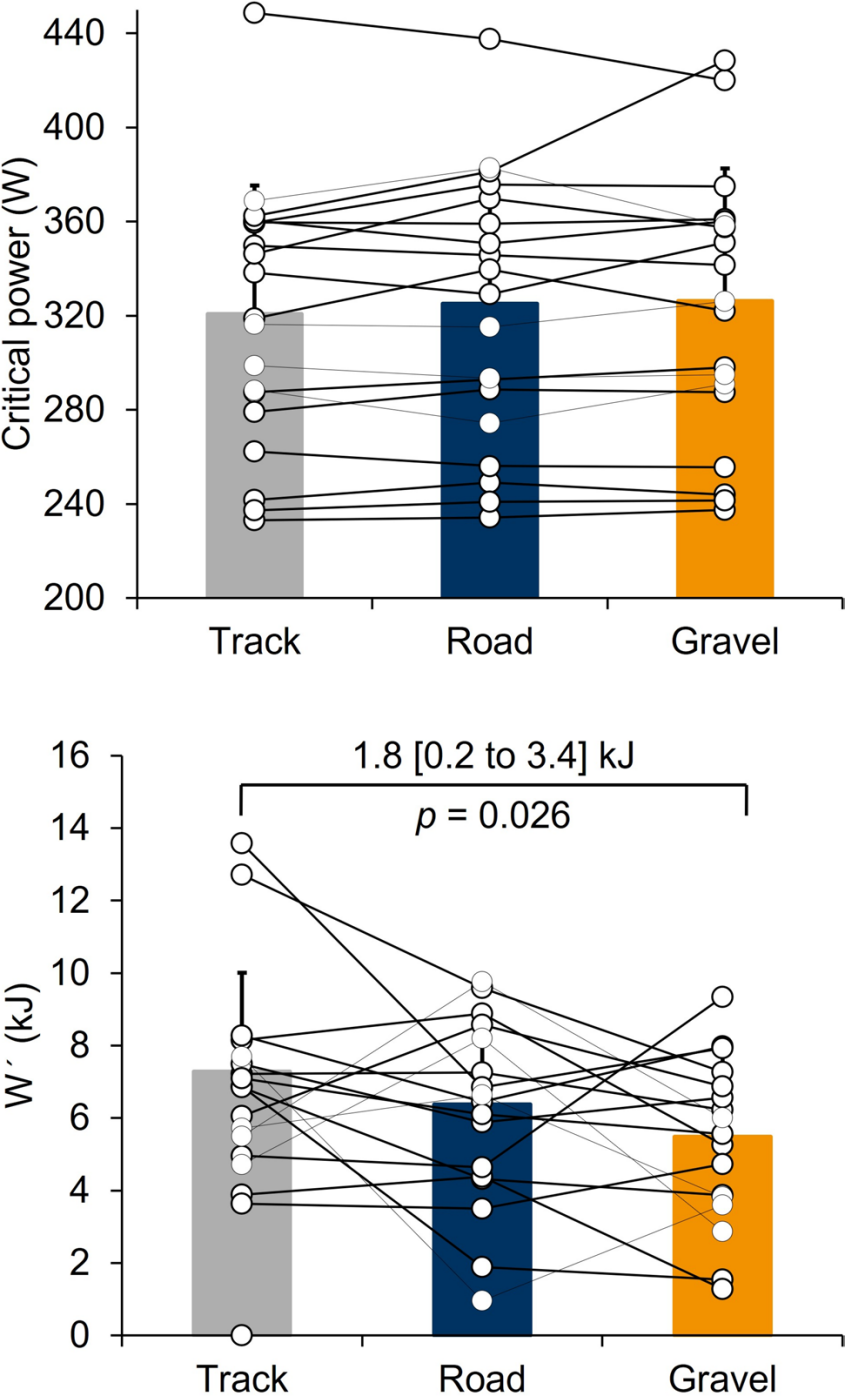
Individual and group effects to track, road, and gravel surfaces on CP and W’. *CP* Critical power, *W’* Work over critical power

Descriptive values of the CP and *W*’ parameters, together with power output, speed, and distance covered in each running bout displayed on each surface are provided in Table 2. There were no significant differences in CP across the different surfaces (*F*_(2,38)_= 1.32; *p* = 0.279). However, significant differences were found in *W*’ (*F*_(2,38)_= 3.8; *p* = 0.032). Specifically, athletes displayed a higher *W*’ in track compared to gravel (1.8 [0.2–3.4] kJ, *p =* 0.026), and higher, though non-significant, *W*’ on the road compared to gravel (0.9 [−0.7 to 2.5] kJ, *p* = 0.478) (Figure 2).

**Table 2.** Influence of track, road, and gravel surfaces on the CP and running performance variables during the 9- and 3-min running bouts

	Track	Road	Gravel	SWC	ANOVA	*η*^2^*p*
CP variables						
CP (W)	321 ± 55	325 ± 57	323 ± 53**$	11	*F*_(2,38)_= 1.32 *p* = 0.279	0.065
W’ (kJ)	7.6 ± 2.7	5.8 ± 3.4	5.9 ± 3.3$	0.63	*F*_(2,38)_= 3.80 ***p* = 0.032**	0.151
9-min running bout						
Power (W)	334.6 ± 57.4	335.7 ± 59.1	334.7 ± 55.9	11.5	*F*_(2,38)_= 0.208 *p* = 0.813	0.011
Speed (km/h)	17.28 ± 1.79	16.79 ± 1.70*	16.53 ± 1.54**$	0.34	*F*_(2,38)_= 13.9 ***p* < 0.001**	0.42
Distance (m)	2621.18 ± 273.80	2524 ± 262.13*	2484.24 ± 238.21**$	51.61	*F*_(2,38)_= 17.8 ***p* < 0.001**	0.48
3-min running bout						
Power (W)	363 ± 64	360 ± 66*	355 ± 63.2$	12.88	*F*_(2,38)_= 2.51 *p* = 0.095	0.12
Speed (km/h)	18.82 ± 1.70	18.28 ± 1.76*	18.21 ± 1.70$	0.34	*F*_(2,38)_= 8.73 ***p* < 0.001**	0.31
Distance (m)^	967.3 (164.34)	906.7 (162.25)*	908.3 (114.09)$	21.63	*Χ*^2^_(2)_ = 15.1 ***p* < 0.001**	>0.55

*CP* Critical power, *W*´ Work over critical power, *SWC* smallest worthwhile change, *ANOVA* Analysis of variance, *η*^2^*p* partial eta squared, *W* watts, *kJ* kilojoules, *km/h* kilometre per hour, *m* metre

Significance is set at *p* < 0.05

^ Data are median (IQR) and *p* values are calculated via Friedman test, pairwise comparisons (Durbin–Conover). Effect size is calculated via Kendall’s *w*

* Track vs Road

** Road vs Gravel

$ Track vs Gravel

During the 9-min running bout, mean power output did not differ significantly between surfaces (*p* = 0.813). However, both running speed and distance covered showed significant differences (*p* < 0.001). Specifically, speed and distance were significantly higher on the track compared to the gravel (*p* < 0.05), and speed was also higher on the road than on the gravel.

### Running pattern variables

**Fig. 3:**
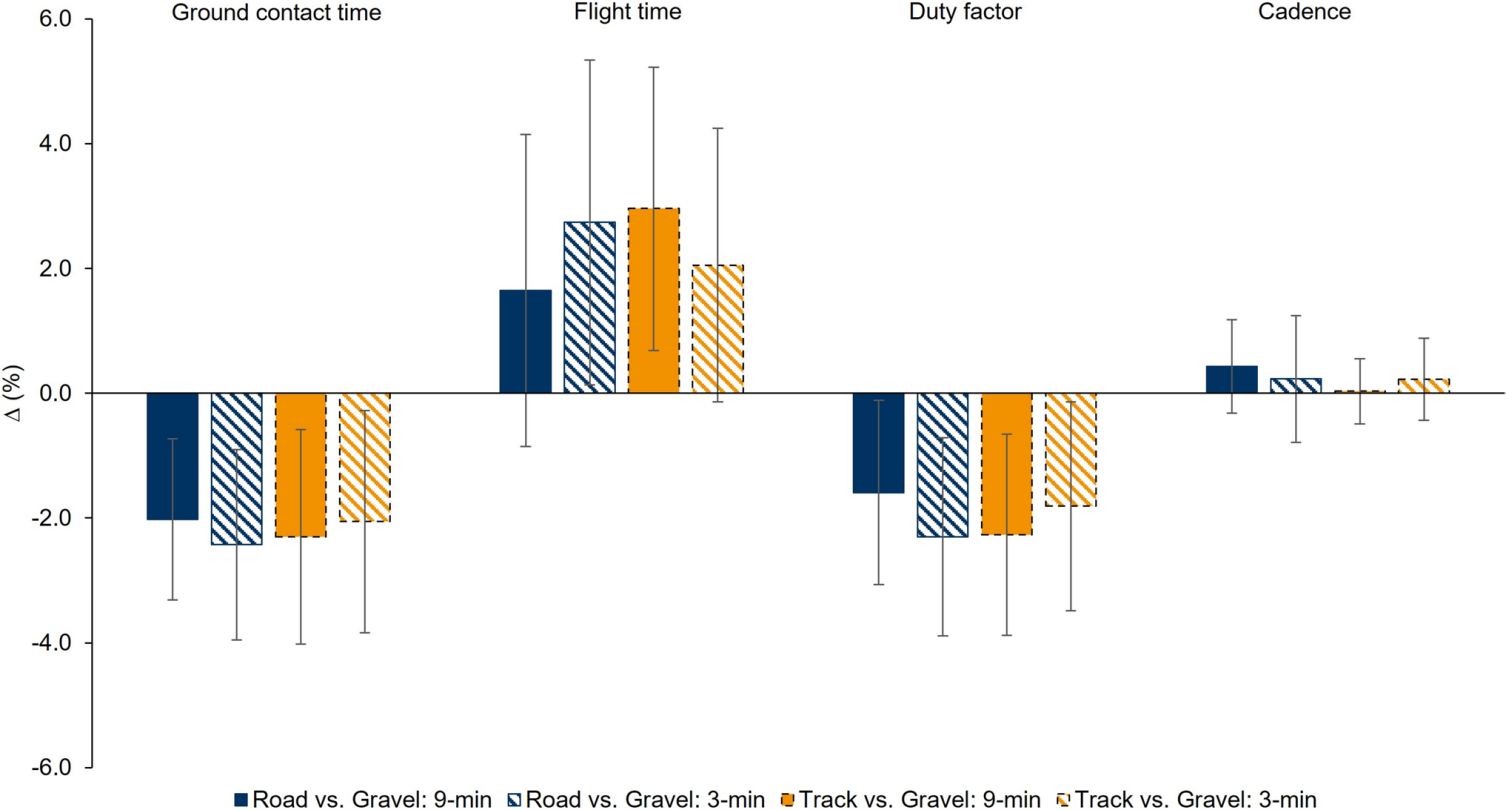
Changes in running pattern variables on track and road with respect to gravel surface. *∆* (%) = (Track or road–gravel)/Mean [Track or road and gravel])*100

**Fig. 4:**
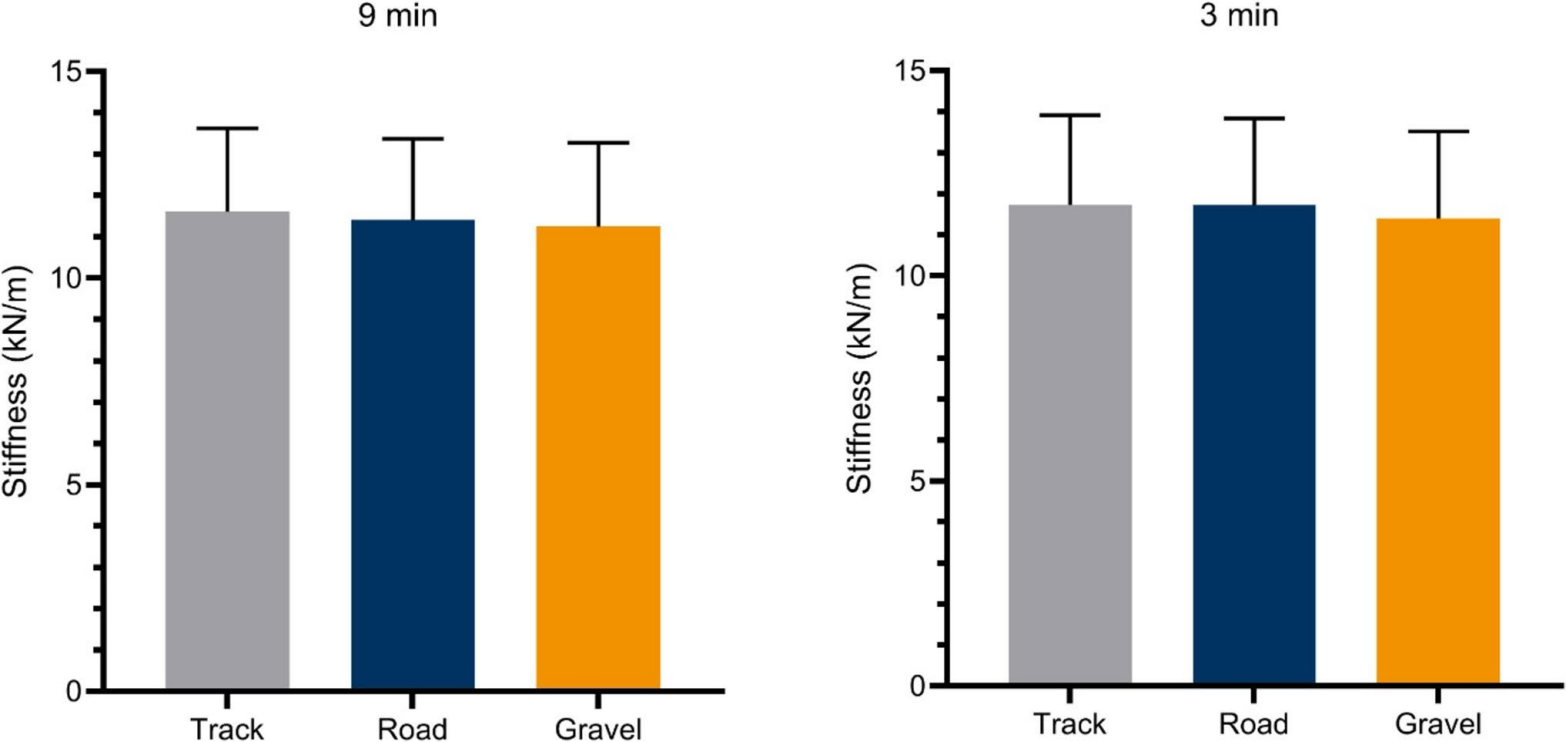
Leg stiffness comparison between track, road, and gravel for the 9- and 3-min running bouts

Descriptive values of the GCT, FT, cadence, DF, and leg stiffness displayed in the running trials (i.e. 9- and 3-min) performed at each surface are provided in Table 3. There was a significant main effect on GCT (*F*_(2,38)_= 7.2; *p* = 0.002), FT (*F*_(2,38)_= 4.2; *p* = 0.021) and DF (*F*_(2,38)_= 6.6; *p* = 0.003). Specifically, athletes displayed a shorter GCT on track compared to gravel (−3.9 [−7.5 to −0.4] ms; *p* = 0.026) as well as on road compared to gravel (−3.9 [−6.6 to −1.2] ms; *p =* 0.003). Athletes displayed longer FT on track compared to gravel (3.5 [0.0 to 7.0] ms; *p =* 0.049), and longer but not significantly different FT on road compared to gravel (3.1 [−0.7 to 6.9] ms; *p =* 0.134). Athletes displayed lower DF on track compared to gravel (−1.1 [−2.2 to −0.1] %; *p* = 0.030) as well as on road compared to gravel (−1.1 [−2.0 to −0.1] %; *p =* 0.019). No significant interactions were reported (*F*_(2,38)_ ≤ 1.3; *p* ≥ 0.279) (Figure 3). No significant differences were found for cadence between surfaces, as well as for leg stiffness (Figure 4). Similarly, no interaction was found for both variables (Table 3).

**Table 3.** Influence of track, road, and gravel surfaces on running pattern variables (mean ± SD) at efforts exerted at the severe intensity domain

	Track		Road		Gravel		ANOVA	
	9-min	3-min	9-min	3-min	9-min	3-min	Main effect	Interaction effect
GCT (ms)	188 ± 21	176 ± 19	188 ± 21	176 ± 19	192 ± 19	180 ± 20	*F*_(2,38)_= 7.2 *p* = 0.002	*F*_(2,38)_= 0.3 *p* = 0.719
FT (ms)	142 ± 19	143 ± 18	140 ± 19	144 ± 19	138 ± 19	140 ± 18	*F*_(2,38)_= 4.2 *P* = 0.021	*F*_(2,38)_= 2.1 *P* = 0.138
Cadence (spm)	182 ± 7	188 ± 8	183 ± 8	188 ± 9	182 ± 8	188 ± 9	*F*_(2,38)_= 0.416 *p* = 0.663	*F*_(2,38)_= 0.454 *p* = 0.639
DF (%)	57 ± 5	55 ± 5	57 ± 5	55 ± 5	58 ± 5	56 ± 5	*F*_(2,38)_= 6.6 *p* = 0.003	*F*_(2,38)_= 1.3 *p* = 0.279
Leg Stiffness (kN/m)	11.6 ± 2	11.7 ± 2.2	11.4 ± 2	11.7 ± 2.1	11.2 ± 2	11.4 ± 2.1	*F*_(2,38)_= 1.6 *p* = 0.211	*F*_(2,38)_= 1.2 *p* = 0.318

*GCT* Ground contact time, *ms* milliseconds, *FT* Flight time, *spm* steps per minute, *DF* Duty factor, *%* percentage, *kN/m* kilonewtons per metre, *ANOVA* Analysis of variance

## Discussion

This study aimed to assess the effects of three distinct flat running surfaces (athletic track, asphalt road, and gravel) on the CP variables and the running patterns of highly trained trail runners. The results revealed that CP remained stable across surfaces, whereas *W*’ was reduced on gravel compared to track and road. The differences in *W*’ were accompanied by significant changes in the athletes’ running patterns. Specifically, athletes exhibited shorter GCT and longer FT on track and road compared to gravel, resulting in a more aerial running style (i.e. lower DF), although their cadence was not significantly altered. Contrary to our initial hypothesis, leg stiffness remained unchanged across the different running surfaces.

The effect of surface on the CP variables has received little attention. A first approximation was seen in the study by Ruiz-Alias et al. (Ruiz-Alias et al. 2023a) where highly trained athletes performed the same CP test used in the present study on the track and treadmill, showing lower absolute power outputs. Although it is expected that such power output differences could rely on some running pattern changes, no spatiotemporal parameters (i.e. GCT and FT) were reported. Furthermore, it was stated that both CP and *W*’ variables should not be extrapolated from the track to the treadmill and vice versa, a practical take-home message for athletes and practitioners when training and testing in indoor and outdoor settings. Deepening this methodological message for running power meter users, it could be added that the flat surfaces here evaluated do not influence the athletes´ intensity prescription (i.e. CP), but performance might be hindered on high-intensity efforts exerted over CP (i.e. *W*’). The results of this study revealed that although running speed and distance covered significantly decreased from track to road and gravel surfaces, mechanical power output remained relatively stable across conditions, with only non-significant differences observed. This finding supports previous research (Cerezuela-Espejo et al. 2021) suggesting that mechanical power is more tightly linked to metabolic power, and thus overall physiological effort, than external indicators such as speed or distance. Notably, CP did not vary significantly across surfaces, highlighting the limitations of speed-based metrics when prescribing or monitoring training load under varying environmental conditions. The stability of mechanical power across different terrains suggests that despite reduced running speed and distance, the metabolic demand imposed on the athlete remains comparable. This is particularly relevant for training prescription in outdoor environments, where surface conditions may vary but the desired training stimulus can be maintained by targeting power-based thresholds. Additionally, the increasing trend in GCT from track to gravel may explain the lack of significant power differences despite decreased running speed. Gravel surfaces, due to their compliant and uneven nature, likely require longer ground contact to achieve similar force production, implying that runners adapt by altering their neuromechanical strategies rather than increasing mechanical output. This highlights the role of surface-specific biomechanical adaptations, particularly in the context of the stretch-shortening cycle, where energy storage and return are modulated by surface stiffness and contact dynamics (Moritz and Farley 2005; Márquez et al. 2014). Overall, these findings reinforce the utility of running power as a more stable and physiologically relevant parameter than speed for quantifying and comparing effort across diverse running environments.

The observed differences in running pattern variables across surfaces offer further insight into the underlying biomechanical and neuromuscular adjustments that accompany changes in running performance and power output. Notably, ground contact time (GCT) was significantly shorter on track and road compared to gravel, while flight time (FT) was significantly longer on the track than gravel. These findings suggest a shift towards a more efficient stretch-shortening cycle (SSC) on harder surfaces, characterized by reduced ground interaction and increased aerial time. This is consistent with the observed higher W′ values on the track, which reflect a greater capacity for work above critical power and have been linked in prior research to more aerial running patterns (Ruiz-Alias et al. 2023c). The lower DF on track and road also supports this interpretation, reinforcing the idea that runners adopt a more elastic and efficient gait on firmer surfaces. Likewise, our results are in congruence with the ones provided by Hollis et al. (Hollis et al. 2019). In this study, a group of recreational runners completed two 1600m at slow and fast self-selected paces at track and grass surfaces, displaying in GCT significant differences at fast but not slow paces, similar to our GCT differences between track, road and gravel, and the observed difference on W´ but not on CP. Of note, despite changes in GCT, FT, and DF, neither cadence nor leg stiffness differed significantly across surfaces. This may be explained by the lack of change in cadence, a key determinant of leg stiffness (Jaén-Carrillo et al. 2022), and by the fact that the current study involved highly trained athletes running at severe intensities, conditions under which stiffness may be preserved across varied surfaces due to superior neuromuscular control. This contrasts with previous findings in less trained populations and under low-to-moderate intensities (i.e. 10–12 km/h) (Ferris et al. 1999; Mohr et al. 2023). Additionally, the running surfaces investigated in previous research differ from those used here. Ferris et al. (Ferris et al. 1999) compared a "soft" and a "hard" surface, while Mohr et al. (Mohr et al. 2023) examined an asphalt road and a woodchip track, surfaces that are less commonly used by runners. In contrast, our use of ecologically valid surfaces (i.e. track, asphalt, and gravel) (Hamill et al. 2022) enhances the applicability of these findings to real-world training environments. Taken together, the biomechanical adjustments observed here likely reflect surface-specific strategies to maintain metabolic power output and optimize performance, particularly in the context of high-intensity endurance running.

Lastly, some limitations and future perspectives should be recognized and discussed. First, it should be noted that athletes wore their own running shoes to complete the different CP tests. In this regard, certain footwear features such as the sole design may interact with the surfaces, particularly on gravel, where specific trail soles are expected to address traction limitations. Nevertheless, all of them always used the same footwear on the three different surfaces. The present study assessed running performance on flat terrains. It is well known that trail running courses typically involve varying gradients of ascents and descents. While in ascents, the Stryd power output appears to align with the athletes’ internal load (Hingrand et al. 2023), during descents, it does not seem to be the case according to the athletes’ perceived effort, which warrants further investigation. Also, further investigation is required to analyse whether the adoption of specific running shoes (track, road, and trail running shoes) would affect the findings here reported.

## Practical applications

This study offers valuable insights for athletes and coaches regarding training surface selection and its impact on performance. While CP remains consistent across flat surfaces, W´, a measure of performance potential above CP, is reduced on gravel due to changes in running patterns. Practically, this suggests that while intensity prescriptions based on CP may remain stable across different surfaces, high-intensity efforts (related to W´) may be compromised on less stable surfaces where traction may be hindered like gravel. Coaches and athletes can use this information to strategically choose training surfaces based on session goals, favouring stable, hard surfaces like track or asphalt to maximize W´-related adaptations, while gravel may be better suited for conditioning, stabilization, and trail-specific training, being sure that CP values still reflect the prescribed running intensity in the three different surfaces. The calculated SWC values provide a practical benchmark for interpreting the magnitude of performance differences observed across running surfaces. For instance, the SWC for critical power (11.0 W) and W′ (0.63 kJ) suggests that only changes exceeding these thresholds are likely to be meaningful in terms of training adaptation or competitive performance. Similarly, in the 9- and 3-minute running bouts, differences in power output (SWC = 11.5–12.88 W), speed (SWC = 0.34 km/h), and distance (SWC = 21.63–51.61 m) help contextualize the relevance of statistically significant findings. While some pairwise comparisons were statistically significant (e.g. speed and distance between track and gravel), not all observed changes across surfaces exceeded the corresponding SWC thresholds, suggesting that certain differences, although statistically significant, may have limited practical impact. These findings underscore the importance of considering both statistical and practical significance when evaluating performance outcomes in endurance running across variable terrain. Low-cost, user-friendly wearable technology facilitates broader implementation of the findings here reported beyond controlled lab settings. Additionally, the findings emphasize the importance of neuromuscular factors (e.g. GCT) in achieving a more aerial running style, which has been linked to better performance at higher intensities. Consequently, incorporating exercises to improve neuromuscular efficiency may be beneficial for trail runners aiming to optimize their performance.

## Conclusion

This study examined the effects of different flat running surfaces on CP variables and running patterns in highly trained trail runners. CP remained stable across surfaces, while W’ was reduced on gravel, coinciding with longer GCT, shorter FT, and a less aerial running style. Contrary to expectations, leg stiffness was unchanged. These findings suggest that while CP can guide intensity prescription across surfaces, high-intensity efforts above CP (W’) may be affected by surface conditions. The use of wearable technology demonstrated practical field applicability. Future research should explore the impact of specific footwear and variable-gradient courses. These insights help athletes and coaches optimize training and competition strategies based on running surface characteristics.

## Conflict of interest

The authors report no conflict of interest.

## Data Availability

The dataset supporting this study is available from the corresponding author upon reasonable request.
